# Targeting Transcription Factors for Cancer Treatment

**DOI:** 10.3390/molecules23061479

**Published:** 2018-06-19

**Authors:** Mélanie Lambert, Samy Jambon, Sabine Depauw, Marie-Hélène David-Cordonnier

**Affiliations:** INSERM UMR-S1172-JPARC (Jean-Pierre Aubert Research Center), Lille University and Hospital Center (CHU-Lille), Institut pour la Recherche sur le Cancer de Lille (IRCL), Place de Verdun, F-59045 Lille, France; melanie.lambert@inserm.fr (M.L.); samy.jambon@inserm.fr (S.J.); sabine.depauw@inserm.fr (S.D.)

**Keywords:** transcription factor, inhibitors, oncogenes, DNA binding, protein/DNA interaction, protein/protein interaction

## Abstract

Transcription factors are involved in a large number of human diseases such as cancers for which they account for about 20% of all oncogenes identified so far. For long time, with the exception of ligand-inducible nuclear receptors, transcription factors were considered as “undruggable” targets. Advances knowledge of these transcription factors, in terms of structure, function (expression, degradation, interaction with co-factors and other proteins) and the dynamics of their mode of binding to DNA has changed this postulate and paved the way for new therapies targeted against transcription factors. Here, we discuss various ways to target transcription factors in cancer models: by modulating their expression or degradation, by blocking protein/protein interactions, by targeting the transcription factor itself to prevent its DNA binding either through a binding pocket or at the DNA-interacting site, some of these inhibitors being currently used or evaluated for cancer treatment. Such different targeting of transcription factors by small molecules is facilitated by modern chemistry developing a wide variety of original molecules designed to specifically abort transcription factor and by an increased knowledge of their pathological implication through the use of new technologies in order to make it possible to improve therapeutic control of transcription factor oncogenic functions.

## 1. Introduction

More than 2500 human proteins associated with different biological processes such as DNA replication, DNA repair, chromosome condensation or DNA unwinding and of course DNA transcription, are thought to bind to chromatin. A very large proportion of these DNA-interacting proteins (~1500) are presumed to act as transcription factors. Transcription factors are proteinsthat bind DNA helix at specific regulatory sequences in orderto activate or inhibit transcription through a transactivation or trans-repression domain. In all living organisms, the ranscription process leads to the expression of ribonucleic acids (mRNA, rRNA, tRNA, lnc-RNA, MiR…) in a fine and spatiotemporally controlled manner and is activated by external or inernal stimuli through a complex signaling network.The transcription factors are organized in different families reflecting homologies in their DNA-binding domains and, consequently, DNA-binding sequences [1,2]. They could be classified in 71 different families with some of them having a larger number of members than the others, such as zing-finger C2H2 (>600 members), homeobox (>200 members) and HLH (>80 members) families that represent >50% of the total number of transcription factors [3]. There is more and more implication of transcription factors in human pathologies in the literature. Indeed, in 2009, Vaquerizas et al. identified 164 transcription factors (~12%) as directly implicated in 277 diseases [3]. A more recent evaluation results in the implication of a larger number of transcription factors in human diseases. For instance, by crossing the list of 1571 known and candidate oncogenic proteins from the Network of Cancer Genes NCG5.0 (http://ncg.kcl.ac.uk/statistics.php) [4] with the list of the 1988 human transcription factors and regulators [5], a list of 294 oncogenic transcription factors and regulators is found (Table 1), corresponding to ~19% of all known oncogenes. 

Besides house-keeping transcription factors, some other transcription factors, as many other class of proteins, are spatially, temporally and sequentially expressed in tissues during development, cell renewal or differentiation processes; and any modification of their expression may result in master deregulation of cell integrity or organism homeostasis leading to pathologies. This is the case for neurodegenerative pathologies [6,7,8,9,10], diabetes or cardiac diseases [11,12,13] and cancers [13,14,15,16,17,18,19,20,21] with either direct implication of transcription factors/repressors or epigenetic modifications of the physical accessibility of specific genomic regions occurring after genetic alterations. Besides some transcription factors are clearly associated with oncogenic addiction [22], only a small number are currently targeted in clinic. Indeed, transcription factors were for a long time considered as ‘undruggable’ targets [23]. A better knowledge of their precise functions (expression, degradation pathway, protein/protein interaction) and the dynamic of their mode of binding has changed this postulate and opened new possibilities to affect transcription factors as therapeutic targets for cancer treatment. Among the different opportunities to indirectly or directly target a transcription factor are:

- Their inhibition (or activation) at the expression level,
- Their inhibition through physical degradation,
- Their inhibition (or activation) at the protein/protein interaction level,
- Their inhibition (or activation) through the binding of a ligand-based molecule in an activation/inhibition pocket,
- Their inhibition (or activation) at the protein/DNA binding level.

All those approaches are developed in the following sections.

## 2. Targeting Transcription Factor at the Expression Level

The expression of a transcription factor of interest, like all other cellular proteins, is itself transcriptionally controlled by transcription activators or repressors (other transcription factors or itself in a positive or negative retro-control) and by epigenetic DNA or histone writers/readers/erasers.

Epigenetic writers modify the DNA or histones by adding for instance methyl, acetyl, ubiquine, SUMO or phosphate groups. Among them are histone methyltransferases that methylate lysine or arginine residues (protein lysine methyltransferases (PKMTs) and protein arginine methyltransferases (PRMTs)), histone acetyltransferases (HATs) that transfer an acetyl group from the acetyl-CoA co-factor to lysine residues on histone tails, the E1/3 unbiquitin ligases and DNA methyltransferases (DNMTs).

Epigenetic readers recognize the epigenetic marks and lead to activation or repression of the transcription process. Among them are bromodomain-containing proteins (BCPs) such as BRD4, and ARID1A.

Epigenic erasers remove epigenic marks added on DNA or histones by the writers. Among them are histones demethylases such as the lysine-specific demethylase 1 (LSD1) and Jumonji C domain-containing demethylases (JMJD), histone deacetylases (HDACs), deubiquitinating enzymes (DUBs) and histone phosphatases. DNA methylation erasers are for instance the gene silencing family of ten-eleven translocation proteins (TETs), catalyzing the conversion of 5-methyl-cytosine to 5-hydroxymethyl-cytosine, or the activation-induced cytidine deaminase (AID). 

### 2.1. Example of HOXA Cluster Expression Controlled by MLL Complex

**Figure 1:**
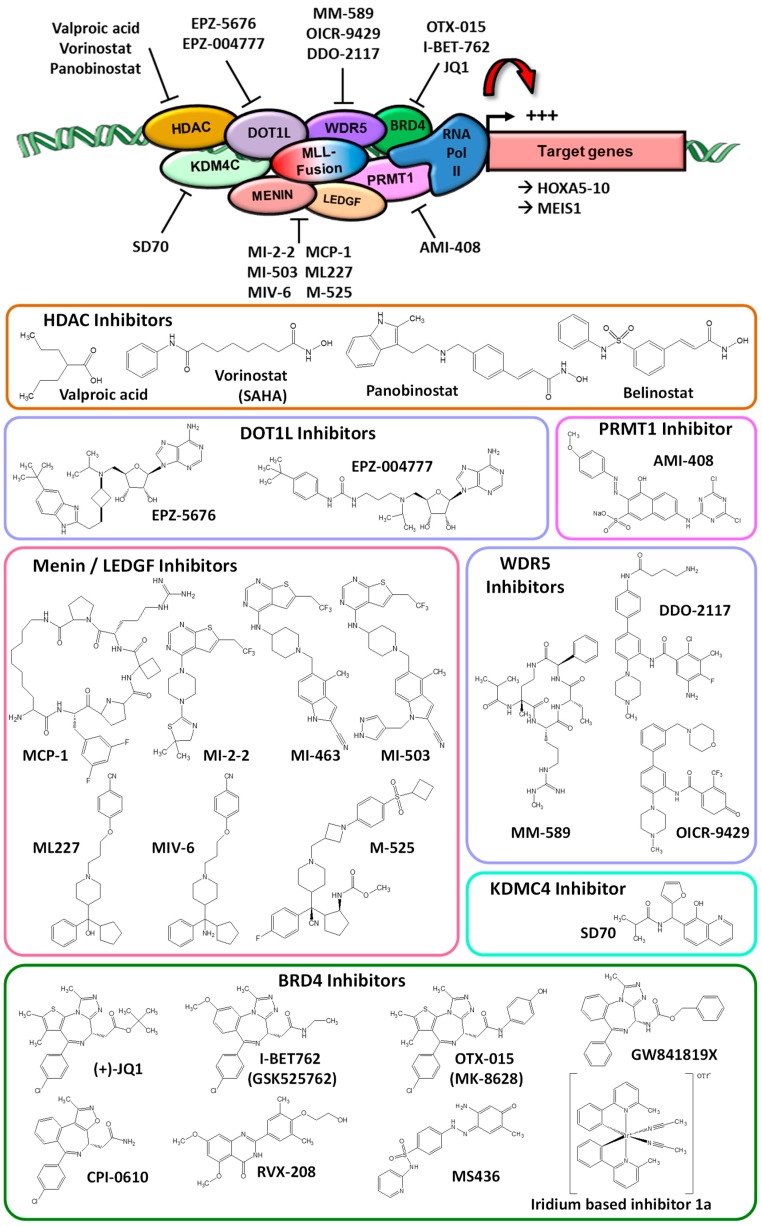
Targeting the MLL complex at the epigenetic level controls the expression of the HOXA cluster of transcription factors. Top of the figure: scheme of proteins associated with the fused-MLL complex highlighting the ones targeted by the different inhibitors indicated below.

Such control of transcription factor expression in cancer treatment is particularly well illustrated in the model of HOXA cluster of transcription factors which is aberrantly expressed under the control of the MLL complex in the context of leukemia. Indeed, MLL proteins are mutated or fused to multiple partners in all mixed-lineage leukemia corresponding to ~5% of acute myeloid leukemia (AML) and ~20% of acute lymphoblastic leukemia, leading to the positive control of HOXA5-10 proteins [24]. Oncogenic MLL fusions or mutated proteins interact with different co-factors associated in the “MLL complex”, most of them associated with epigenetic control. Among them are HDAC [25,26], DOT1L [27,28,29], BRD4 [30], Menin [31,32], LEDGF [31,33], WDR5 [34], KDM4C (JMJD2C) and PRMT1 [35] (Figure 1). Many of them are targeted for cancer treatments (for review: Steinhilber 2017) [36].

This is the case of HDAC inhibitors such as valproic acid, vorinostat (suberoylanilide hydroxamic acid, SAHA, Zolinza^®^), the cinnamic hydroxamic acid analogue panobinostat (LBH589, Farydak^®^) or belinostat (PXD101, Beleodaq^®^) (Figure 1) that entered into clinical trials in AML [37,38,39,40] and were approved in other leukemia or hematological diseases such as vorinostat for the treatment of cutaneous T cell lymphoma (CTCL), panobinostat against multiple myeloma or belinostat to treat peripheral T-cell lymphoma [41]. However, those marketed HDAC inhibitors are not selective to one or another of the HDAC isoenzymes, leading to major epigenetic disorders associated with off targets deregulations. 

For DOT1L histone methyltransferase (HMT), its aberrant recruitment at MLL complex occurs through the interaction with MLL fusion partners AF9 [42] AF4 [28] AF6 [30], AF10 [43] or ENL [44]. DOT1L could be inhibited using ligand competitors, such as EPZ-5676 (pinometostat) and EPZ004777 (Figure 1), that occupies the *S*-adenosyl-methionine (SAM) binding pocket of DOT1L, resulting in conformational changes that abolish HMT function. The cyclo-butyl derivative EPZ-5676 is more efficient than the parental compound EPZ004777 on DOT1L inhibition (*Ki* of 0.08 nM for EPZ-5676 vs. *Ki* of 0.3 nM for EPZ004777) [45,46]. Consequently, EPZ-5676 was chosen as a first-in-class HMT inhibitor and entered clinical trials in relapsed/refractory AML associated with MLL rearrangements. EPZ-5676 and EPZ004777 subsequently interfere with leukemic process leading to cell death and differentiation [47,48,49,50] and also recently evidence other therapeutic opportunities such as the induction of osteoclast differentiation [51]. 

Menin/LEDGF inhibitors (Figure 1) are another therapeutic option to interfere with pathologic MLL function that control HOXA transcription factors expression. Among them are:

- The macrocyclic peptidomimetic MCP-1 [52],
- The thienopyrimidine MI-2-2 [53] and its derivatives MI-463/503 [54] with MI-2-2 being poorly stable and could not be used in vivo rather than MI-463 and MI-503 (a derivative of MI-463 by the addition of a single methylpyrazole) which both interact with menin at nanomolar range, are more metabolically stable and exert strong cellular and in vivo activity, MI-503 being the most efficient one with deeper contacts with the menin pocket [54],
- The hydroxymethylpiperidines ML227, MIV-6 and cyclopentylphenylpiperidine derivative M-525 [55,56,57] that mimic the interacting MLL peptide and may be used together with DOT1L inhibitors to restore differentiation in MLL-rearranged leukemias [58]. ML227 presents poor metabolic stability as well as off target activities that limited its developement and an IC_50_ for interation to menin of 390 nM [56]. MIV-6 differs from ML227 by an amine group that substitute to the hydroxyl group of ML227 and is more stable but with similar range of IC_50_ for menin (185 nM) whereas M-525 is much more efficient on menin interaction with IC_50_ of 3.3 nM and is 30-fold more potent in cellular activities with a hiogh specificity on mixed lineage leukemia cell models such as MV4;11 [57]. Inhibitors of BRD4 also showed efficiency to target mutated MLL functional complex, based on their interaction to control gene expression [59,60] and to collaborate with DOT1L [61]. This is the case for the thienodiazepine (+)-JQ1, I-BET762 (GSK525762), OTX015, GW841819X, CPI-0610 and RVX-208 that are developed by different companies and entered into clinical trials in various hematological malignancies and solid tumors while other compounds such as, MS436 or the iridium based inhibitor **1a** (Figure 1) are in developmental stages (for reviews Huang 2016; Liu 2017; Kharenko 2017) [62,63,64]. Moreover, it is worth noting that both BRD4 and DOT1L inhibitors could synergistically inhibit proliferation of MLL-rearranged leukemic cells [61].

Recently, inhibitors of WDR5/MLL interaction were also developed such as the macrocyclic MM-589 compound [65,66] or DDO-2117 and OICR-9429 [67,68], as well as AMI-408 [69] and SD70 [70] that respectively inhibits the H4R3 methyltransferase PRMT1 and jumonji domain-containing H3K9 demethylase KDMC4, two proteins associated with oncogenic MLL complex as well described for MLL-GAS7 translocation [35]. 

Altogether, the different protein partners of MLL and their inhibitors summarized in Figure 1 encompass the therapeutic opportunities to control HOXA5-10 transcription factor at the expression level through deregulated-MLL complex. But HOXA9 transcription factor expression may also be controlled by other epigenetic modifiers such as (i) inhibitors of the epigenetic eraser H3K4 demethylase LSD1/KDM1A [71,72] like GSK2879552 [73] and ORY-1001 [74,75] that induce leukemic cell differentiation and are currently in clinical trials; (ii) inhibitors of the H3K9 methyltransferase G9A/KMT1C (UNC0648) [76] or inhibitors of the H3K27 methyltransferase EZH2 (GSK126, UNC1999, CPI-1205, EPZ005687, EPZ-6438/tazemetostat) [77,78,79,80,81,82] as two well described epigenetic writers associated with leukemia.

### 2.2. Example of MYC Transcription Factors Expression Control at the Epigenetic Level 

**Figure 2:**
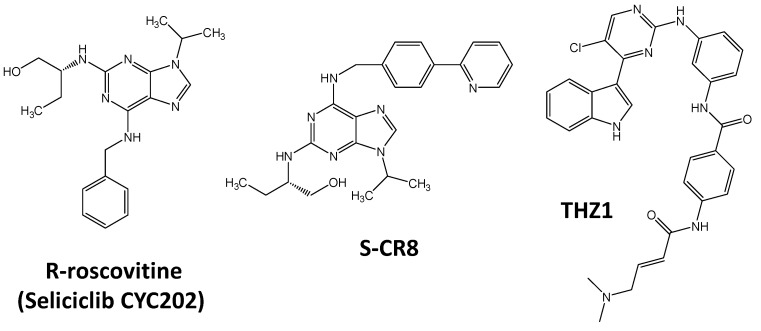
CDK7 inhibitors for targeting MYC transcription factor promoter at the epigenetic level.

Another well-studied oncogene transcription factor family which expression could be epigenetically modulated for therapeutic approaches is MYC gene family. Multiple cancer and hematological diseases are associated with c-MYC transcription factor deregulations such as gene amplification, translocations, promoter polymorphism or mutations [83]. For instance, c-MYC gene translocations with immunoglobulin genes, such as t(8;14), t(8;22) or t(2;8), are associated with Burkitt lymphoma, diffuse large B-cell lymphoma, plasmablastic lymphoma, mantle cell lymphoma and in the evolution of pre-malignant MGUS cells into multiple myeloma [84,85]. Translocation may result in the juxtaposition of enhancer sequence to the minimal promoter of c-MYC gene to control c-MYC expression. C-MYC over-expression is also associated with self-renewal of leukemic stem cells, in relation to the hematopoietic stem cell niche [86]. Despite long term knowledge of its oncogenic activity, c-Myc is not yet directly targeted. Only indirect negative control of its expression was developed with inhibitors of BRD4 such as JQ1 or I-BET762 (Figure 1). Indeed, JQ1 displaced BRD4 from c-Myc promoter [87]. Treatment with JQ1 is also associated with cell differentiation as proved by an increase in the macrophage differentiation marker CD74 [88]. HDAC inhibitors such as vorinostat could also control c-MYC gene expression, as shown in T-cell acute lymphoblastic leukemia in vivo models [89,90]. JQ1 could also be used in combination with HDAC inhibitors to repress c-MYC promoter activity [91]. Such epigenetic control is also observed by targeting CDK7 with R-roscovitine (seliciclib, CYC202), S-CR8 or the covalent inhibitor THZ1 (Figure 2) controlling N-Myc gene expression [92,93,94], another Myc family member associated with cancer [95]. 

## 3. Targeting Transcription Factor at the Protein Degradation Level

**Figure 3:**
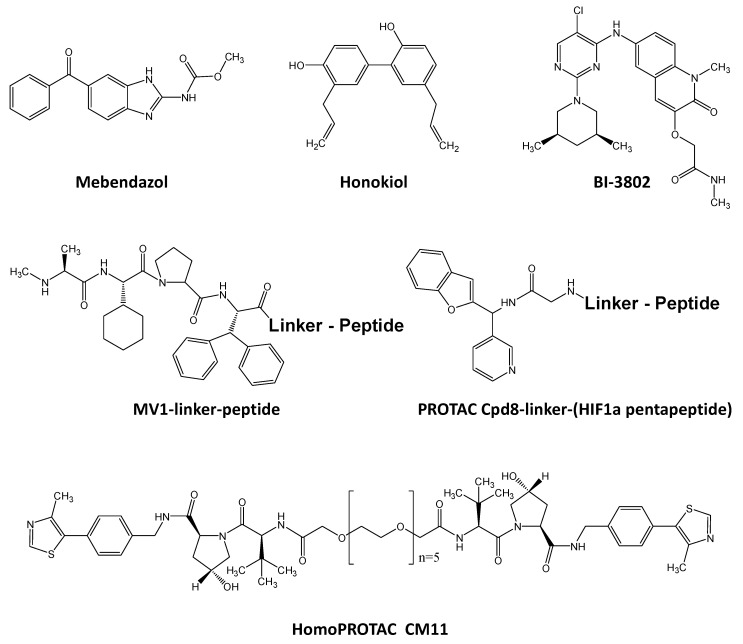
Small compounds (**Top**) or drug-linker peptides (**Middle** and **Bottom**) inducing degradation of oncogenic transcription factors.

Direct transcription factor degradation is another therapeutic option. Strategies leading to the decrease of already expressed transcription factor by the ubiquitin-proteasome or sumoylation processes with compounds such as bortezomib (Velcade^®^) is already well illustrated in the literature and would not be further explored in this review [96,97,98,99,100]. Proteasome degradation is also implicated in therapeutic process consequently to the chemically-induced disruption of protein/protein interaction as described in Section 4 of the present manuscript and reviews [26]. Besides, some original approaches were more recently depicted such as the induction of Myb transcription factor degradation using mebendazole (Vermox^®^) (Figure 3), commonly used to treat a number of parasitic worm infestations [101], or that of the AML1-ETO (RUNX1-ETO) chimeric transcription factor by the natural phenolic compound honokiol (Figure 3) extracted from *Magnolia* spp. [102], both protein targets known to be implicated in acute myeloid leukemia (Figure 3). BI-3802 (Figure 3) inhibits the binding of the BTB domain of BCL6 to co-repressors such as NCOR1 but not the dimerization of the BTB of BCL6 as presented for other inhibitors in Section 4, resulting in functional BCL6 homodimers. However, BI-3802/BCL6-BCL6 trimer complex formation results in subsequent ubiquitinylation and degradation of the BCL6 repressor, leading to an anti-proliferative activity in diffuse large B cell lymphoma and induced expression of BCL6-repressed genes such as ATM, DUSP5 and IRF4, a transcription factor associated with B cell maturation [103]. However, more original aspects are coming from synthesis of ligands coupled to peptides to link specifically targeted transcription factors to proteins associated with degradation. This is the case of hybrids molecules called SNIPERs for Specific and Nongenetic IAP-dependent Protein Erasers such as MV1 compound associated with a peptide derived from MALM1 inhibitory peptide previously developed to inhibit NOTCH1. MV1-linker peptide (Figure 3) stabilizes NOTCH1 transcription factor to the E3 ubiquitin ligase inhibitor of apoptosis protein (IAP) [104]. This is also the case of PROTACs (for PROteolysis TArgeting Chimeric molecules) such as Cpd 8 [105] that associates Smad3 pocket ligand with HIF1 recognition motif of the von Hippel–Lindau (VHL) E3 ubiquitin ligase [106] or CM11 Homo-PROTAC dimer that induced VHL dimerization and sub-sequent self-degradation [107] (Figure 3). 

## 4. Targeting Transcription Factor at the Protein/Protein Interaction Level

Inhibiting transcription factor interaction with other proteins is another approach associated with protein sequestration, stabilization or degradation depending on the nature of the interacting proteins or the context. The partner can be the transcription factor itself forming homo-dimers (STAT, BCL6), another transcription factor (RUNX1/CBFβ, MYC/MAX) from the basal transcription machinery, co-factor/co-activator/mediator or repressor (Nrf2/Keap1), chaperones implicated in nuclear translocation or proteins sequestrating the transcription factor in cytoplasm often associated with degradation process (p53/mdm2). The protein/protein interaction (PPI) inhibitors (PPIi) belong to three classes of molecules: small compounds, peptidomimetics or stapled helix peptides. The following part gives an overview of the different families of molecules targeting different functions of PPI with some examples of targeted transcription factors from different DNA binding sub-families.

**Figure 4:**
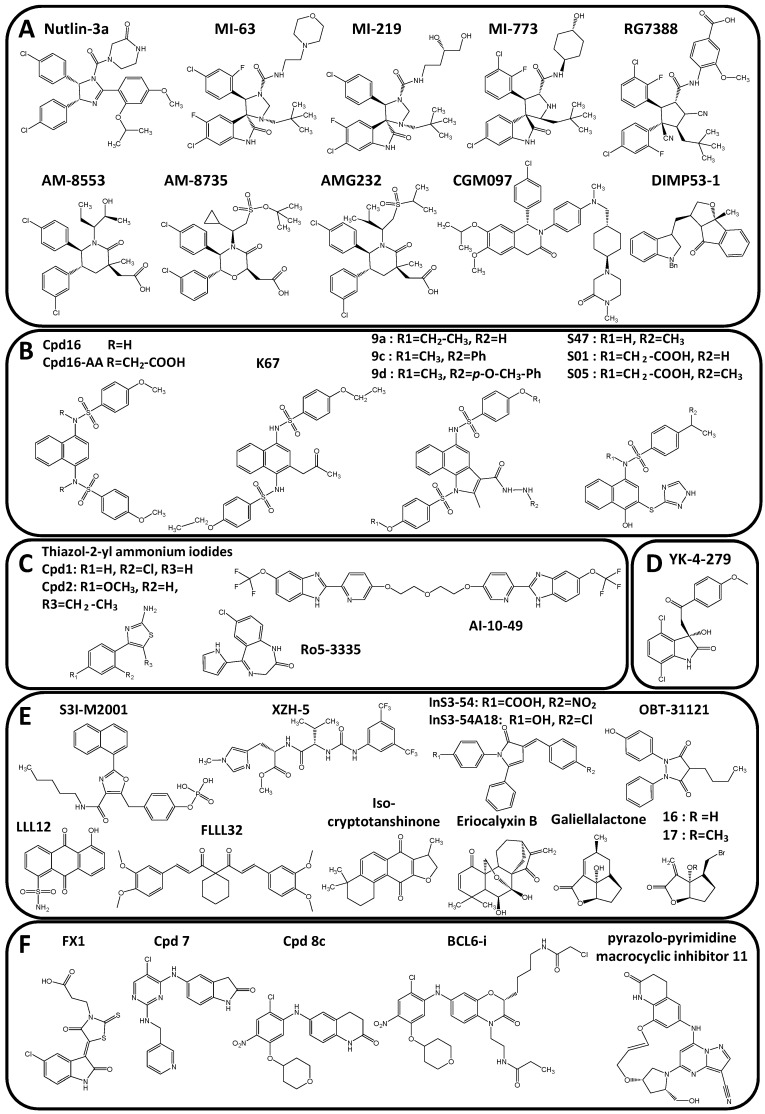
Targeting transcription factor at the protein/protein interaction level. (**A**) p53/mdm2 PPIi; (**B**) Keap1/Nrf2 PPIi; (**C**) RUNX1/CBFβ PPIi; (**D**) Ets transcription factors/RNA helicase A PPIi; (**E**) STAT3 PPIi; (**F**) BCL6 PPIi.

The tumor suppressor transcription factor p53 was the first transcription factor inhibited at the PPI level. The p53 protein binds DNA as a tetramer to control the expression of its target genes among which are PUMA and p21. In cancer, p53 is mutated and/or maintained in the cytoplasmic compartment through interaction with the Murine Double Minute 2 (mdm2) protein, a protein that is over-expressed in around 50% of all cancers [108]. The p53/mdm2 PPI leads to p53 ubiquitinylation by E3 ubiquitin ligase and subsequent proteasomal degradation [109]. The p53/mdm2 PPI is one of the most studied PPI with many PPIi developed for their binding to MDM2 in the p53-binding triad pocket consisting of the three hydrophobic amino acids Phe19, Trp23 (interaction with Leu53 of mdm2) and Leu26. The first compound that entered clinical trial is the chiral (−) Nutlin-3 (Figure 4A) that presents a bromophenyl ring deeply hooked in Leu26 pocket and an ethyl-ether arm interacting with the Phe19 pocket while its imidazoline backbone localizes at position usually taken by the p53 alpha helix from its mdm2-binding domain. Many other p53/mdm2 PPIi (Figure 4A) were then developed with more or less different drug/mdm2 contacts (for recent reviews, Lemos 2016; Nayak 2017; Wang 2017) [110,111,112]. This is the case of spiro-oxindoles derivatives MI-63, MI-219 or MI-773/SAR405838 that target Phe19 and Leu26 pockets [113,114,115], the pyrrolidine analogue RG7388 which interacts with Phe19, Trp23, Leu26 and His96 [116], piperidinones such as AM-8553 that make contacts with Phe19, Trp23 and Leu26 [117]. Other p53/mdm2 inhibitors are AMG232, as an orally bioavailable derivative of AM-8553 for clinical use [118], the morpholinone AM-8735 [119], or the spiroindolinone RO8994 [120]. CGM097, MK-8242 and DIMP53-1 are other p53/mdmd2 inhibitors also inhibiting the interaction of p53 with mdmx, hdm2 and/or hdmx [121,122]. In parallel with synthetic compounds, peptidomimetics and peptide staples were also designed and evaluated as p53 inhibitors [123,124,125,126] but their development for clinical used is less advanced than that of synthetic drugs.

The nuclear factor erythroid 2-related factor 2 (Nrf2) presents a CNC basic DNA binding domain and is an another example of transcription factor which activity is regulated upon cytoplasmic sequestration and an oncogenic target [127]. Nrf2 regulates the expression of genes associated with oxidative stress, drug efflux pumps and drug metabolizing enzymes (such as NQO1, HMOX, MRP1 and GST isoenzymes), resulting in an inhibition of apoptosis as well as radio- and chemo-resistance [128]. Nrf2 is indeed maintained in the cytoplasm through interaction with dimers of the sensor of oxidative stress Kelch-like ECH-associated protein 1 (Keap1) [129] through DIDLG and ETGE amino acids of Nrf2, two peptide sequences separated by a lysine-rich motif. Keap1 interacts as a monomer with Nrf2 on the high affinity binding motif ETGE leading to the “open” conformation. The interaction as a dimer of Keap1 with both the high affinity (ETGE) and the low affinity (DIDLG) binding motives of Nrf2 results in the “closed” conformation of Keap1/Nrf2 complex which is ubiquitinylated on the intermediate lysine-rich motif by ubiquitin ligase Cullin3, leading to subsequent Nrf2 protein degradation by the proteasome [130]. PPIi were identified to inhibit Keap1/Nrf2 complex formation, based on this Keap1/Nrf2 PPI interface. High-throughput screening and fragment-based drug discovery identified structures from which several PPIi were synthesized and evaluated. This is the case of Cpd16 and its more active derivatives Cpd16-AA and the acetonyl-naphtalene and ethoxy-derivative K67 [131,132] (Figure 4B). K67 is of particular interest in this series due to, first, a better selectivity index for direct binding to Nrf2 rather than to phospho-p62 protein, another protein interacting with Keap1, and, second, to its inhibitory activity on cell proliferation and on resistance to cisplatin and sorafenib from comparison to Cpd16 [132]. Also deriving from Cpd16 were developed benzo[*g*]indoles **1** and its N-substituted hydrazides **9b**, **9c** and **9e [133]** and the hydronaphthoquinones **S01**, **S05** and **S47** [134] (Figure 4B). In particular, the addition of a unique benzo[*g*]indole skeleton in compounds **1**, **9b**, **9c** and **9e** series induces a 5- to 10-fold decrease of Nrf2/Keap1 PPI inhibitory constant *Ki* and 10-fold increase of the compound metabolic stability as evidenced using human liver microsomes, compound **9e** being the most stable compound [133]. The hydronaphthoquinones **S01**, **S05** and **S47** activities were not compared to Cpd16 activity but were evaluated using cellular and in vivo experiments, showing that both **S01** and **S05** induce nuclear translocation of Nrf-2 and quickly activate the expression of target genes such as the heme oxygenase-1 and the NADPH-quinone oxidoreductase 1, reduces LPS-induced pro-inflammatory cytokines such as TNF-α, IL1β and IL-6 and LPS-induced cell death in mice, suggesting that they could be used in cancer chemoprevention [134]. Besides small inhibitory compounds, Ac-LDPETGEFL-OH peptide and c[GQLDPETGEFL] cyclic peptide were developed and evaluated for their ability to bind to Keap1 and induce the expression of Nrf2-controled genes [135]. Those Nrf2 inhibitors would have applications in cancer but also in other diseases associated with oxidative and inflammatory stress such as diabetes, Parkinson and Alzheimer diseases or cardiomyopathies. 

RUNX1 belongs to the *Runt* DNA-binding domain family of transcription factor. RUNX1 binding to its co-factor CBFβ (Figure 4C) could be inhibited by 4-(2′-Chlorophenyl)-thiazol-2-yl- and 5-Ethyl-4-(4′-methoxyphenyl)-thiazol-2-yl ammonium iodides [136], the benzodiazepine Ro5-3335 [137] and the bivalent trifluoromethoxy-benzimidazole-pyridine compound AI-10-49 [138], as evidenced using FRET and ELISA-based assays. Of interest, Ro5-3335 and AI-10-49 are much more efficient than the thiazol-2-yl ammonium iodides on RUNX1/CBFβ binding inhibition and where further evaluated on AML cell lines.

Both adult AML and pediatric acute lymphocytic leukemia (ALL) models could be associated with fused CBFβ or RUNX1 proteins, such as CBFβ-MYH11 (inv(16) AMLs), RUNX1-ETO or TEL-RUNX1 fusion. Human acute leukemia cell lines bearing such translocation are much more sensitive to AI-10-49 than non-CBFβ or non-RUNX1 translocated cell lines. AI-10-49 is of particular interest for treatment of (inv16) AML as it inhibits RUNX1 binding to CSF1R, RUNX3 and CEBPa promoter targets and also strongly increases mice survival in CBFβ-MYH11 murine leukemia cell models by reducing leukemia burden. 

In the Ets-family, the interaction of EWS-FLI1, EWS-ERG and EWS-ETV1 fusion transcription factors (generated after chromosome translocation and associated with Ewing sarcomas) with the RNA helicase A is inhibited by the (*S*)-enantiomer of YK-4-279 (Figure 4D) [139,140]. YK-4-279 has anti-oncogenic activities evidenced in different cellular and animal models in which it induces cell apoptosis and reduces tumor size from Ewing sarcoma xenografts [140,141], leukemia [142] and neuroblastoma [143]. But also in prostate models due to inhibition of ERG and ETV1, YK-4-279 results in a decrease of the expression of the ERG-target genes PLAU, PLAT and ADAM10 and ETV1-target gene MMP13 [144] associated with in vivo anti-tumor activities [145]. YK-4-279 was granted as an orphan drug in Ewing sarcomas by the US Food and Drug Administration (FDA) under the name Efdispro^®^ for EWS-FLI1 Disrupting Protein. However, since YK-4-279 is highly hydrophobic and poorly bioavailable in mice (2–15%), a prodrug or adequate formulation might be necessary to increase its bioavailability.

For the signal transducer and activator of transcription (STAT) family of transcription factors, homo- or heterodimers could be targeted to treat cancer. STAT3, STAT1 and STAT5 are the main targeted STAT transcription factors. For instance, STAT3 is active as a tyrosine-phosphorylated protein interacting with the Src homology 2 (SH2) domain of STAT3 to form homodimers. Besides inhibition of phosphorylation by JAK tyrosine kinase as JAK/STAT signaling inhibitors, another targeting approach is the inhibition of SH2-domain interaction with P-Tyr at the PPI level. The oxazole-based peptidomimetic S3I-M2001 (Figure 4E) mimics and binds the SH2 domain of STAT3 to disrupt active STAT3 homodimers and STAT1/STAT3 heterodimers [146]. XZH-5 and analogues are other examples of small compounds designed to recognize the SH2 domain of STAT3 to inhibit its phosphorylation and subsequently induce cell death in hepatocellular carcinoma and breast cancer cells [147,148]. This is also the case of LLL12 and the FLLL32 curcumin derivatives [149,150] evaluated in hepatocellular carcinoma, or OPB-31121 or OBP-51602 exerting potent anticancer activities in tumor xenografts. Based on their activites at nM range for inhibition of STAT3 dimers, OPB-31121 and OBP-51602 are some of the most promising ones andentered clinical trials in advanced leukemias, myelodysplastic syndromes, multiple myeloma or advanced solid tumors such as hepatocellular carcinoma [151] (Figure 4E). Isocryptotanshinone (ICTS, Figure 4E) was also found to interact with the SH2 domain of STAT3 (but only at IC_50_ ≈ 5 µM) and to subsequently induce apoptosis and autophagy in A549 adenocarcinoma lung model [152]. Aminotetrazole, benzo-[*b*]-thiophene dioxide, dibenzylidenecyclohexanone derivatives and naphthalene-5,8-dione-1-sulfonamide (Figure 4E) were also developed to be selective of the SH2 domain of STAT3 over STAT1 [153,154,155,156]. By contrast, eriocalyxin B (Figure 4E) inhibits STAT3 through a covalent binding to Cys712, that is closed to the SH2 domain of STAT3, as evidenced using LC-MS/MS, in order to block STAT3 phosphorylation and cell apoptosis of lung and breast cancer cells [157]. Galiellalactone, a natural fungal metabolite isolated from the ascomycetes, and its derivatives **16** and **17** (Figure 4E) also interact with STAT3 in order to inhibit its DNA binding and are effective in prostatic and breast cancer cell models [158,159,160]. Galiellalactone was only moderately active on STAT3-mediated luciferase activity with IC_50_ ≈ 5 μM and on cell growth with cytotoxic activities IC_50_ ≈ 10–20 μM against human breast cancer cell lines. This activity is moderatly increased using the halogen-substituted [3.3] bicyclic lactone derivative **16** with an IC_50_ ≈ 10 μM and the angular alkoxy-substituted analogue **17** with an IC_50_ ≈ 6 μM on the same cancer cell models. Despite relatively poor cellular activities, an orally available prodrug of galiellalactone (GPA512) was developed and showed interesting preclinical evaluations in DU145 prostate cancer xenograft model [161]. All together, this large production of STAT transcription factors inhibitors is in agreement with the growing implication of this transcription factor family in cancer. However, first clinical trials of STAT3-targeting drugs have not yet met success but further development of active drugs is important in the litterature and might offer new opportunities for cancer treatment in the future. 

The basic helix–loop–helix leucine zipper transcription factors are another large family of transcription factors. Interaction of the two bHLH transcription factors MYC and MAX is another well described PPI model to be targeted in cancer. Many structures and strategies to target the interaction domain of those long crossed α-helices are already well depicted in recent reviews (NY2267, Mycro1 and Mycro2, 10058-F4 and 10074-G5…), and would not be further described here [162,163,164]. 

BCL6 is a zinc-finger transcription factor overexpressed, translocated or mutated in several lymphomas among which diffuse large B-cell (DLBCL) and follicular (FL) lymphomas [165,166] and in glioma [167]. BCL6 proteins homodimerize through their BTB/POZ PPI domains to form a specific binding pocket in order to be associated with BCOR (B-cells) and NCOR (neuronal cells) cofactors. From the first defined decoy peptide mimicking the SMRT peptide that interacts with BCL6 and inhibits BCL6/SMRT PPI [168], other peptidomimetics were developed with interesting cellular activities and in vivo anti-lymphoma potencies as L-BPI or PR-BPI [169], F1324 [170] or the cyclo-CIYYCV [171]. F1324 is a promising peptidomimetic based on a dissociation constant *K_D_* of 0.57 nM [170]. In parallel, synthetic inhibitors were also developed as the indolin derivatives 79-6 and FX1 [172] and the diphenylamine derivative **7 [173]** with increasing affinity as exemplify by *K_D_* measurement of 129 µM, 7 µM and 78 nM, respectively (Figure 4F). The tetrahydroquinolinone **8c [174]** and the pyrazolo-pyrimidine macrocyclic inhibitor **11** [175] are other BCL-6 PPIi that present strong affinity for BCL6 homodimers (Figure 4F). More recently, the covalent BCL6 inhibitor BCL6-i (Figure 4F) was synthesized as a chloracetamide derivative of compound **8c** to covalently bond Cys53 of the BCL6-BTB domain, resulting in irreversible inhibition of BCL6 function [176].

Besides these transcription factors which are well studied for PPIi, many others are already, or envisaged to be, targeted by peptidomimetics or synthetic inhibitors for cancer treatment (androgen receptor/TIF2, HIF1α/p300-CBP, HOX/PBX, YAP/TEAD) [112,177,178,179,180]. 

However, a transcription factor acting as a monomer or having no known heterodimer or co-factor, cannot be targeted at PPI level. Another option is to directly block its protein/DNA interface occurring though interaction with a pocket within the transcription factor (ligand pocket or a pocket formed in the DNA-binding domain) or through the interaction with the DNA binding sequences to compete for transcription factor DNA binding activity. Both options are presented below.

## 5. Targeting Transcription Factor through a Binding Pocket

### 5.1. Targeting a Ligand-Binding Pocket

**Figure 5:**
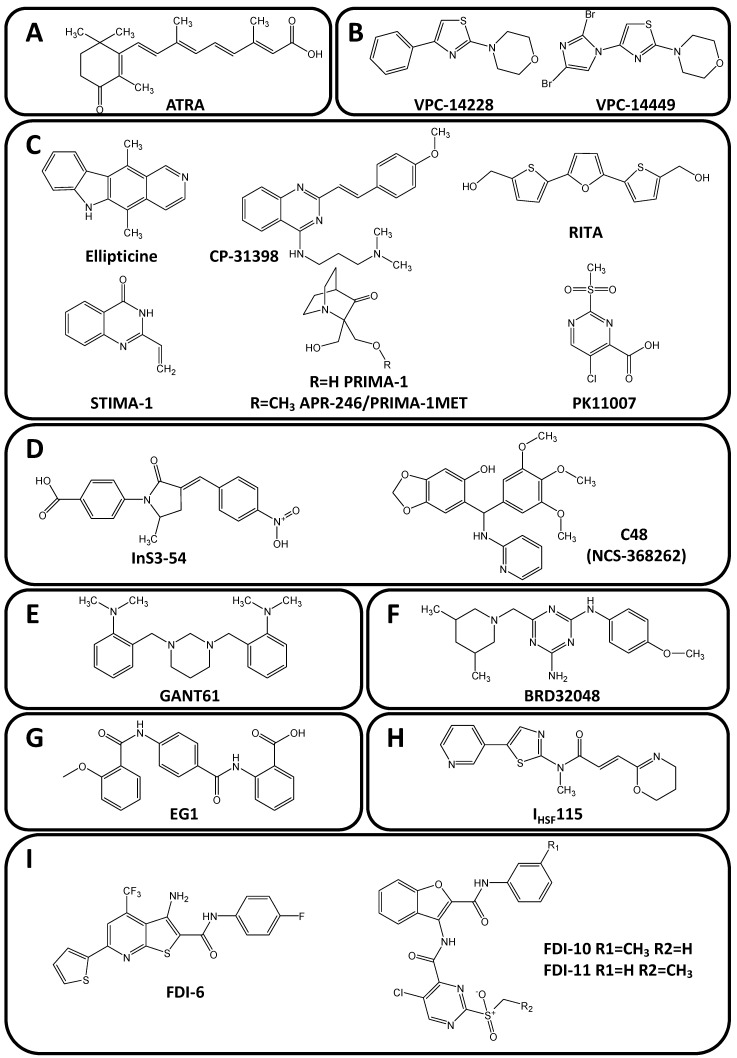
Direct targeting of transcription factors. (**A**) PML-RARα; (**B**) Androgen receptor; (**C**) p53. (**D**) STAT3; (**E**) GLI1/2; (**F**) ETV1; (**G**) PAX2; (**H**) HSF1; (**I**) FOXM1.

The easiest and oldest approach for inhibiting transcription factors through a binding pocket is the development of ligand-derived drugs, taking advantages of an already identified ligand-binding pocket to develop structural derivatives of the natural ligands. This is the case for steroid and hormonal receptors. Some of them are already targeted in cancer treatment for the property of a drug, deriving from their ligand structure, to modulate their DNA binding properties. This is particularly well depicted for the direct binding of all*-trans*-retinoid acid (ATRA, Figure 5A) to PML-RARα oncogenic fusion transcription factor expressed following t(15;17)(q22;q12) translocation which is responsible for acute promyelocytic leukaemia (APL) [181]. ATRA binds to the retinoid ligand pocket of RARα and subsequently induces the dissociation of bound co-repressors. All this leads to PML-RARα degradation to restore binding of the wild-type RARα transcription factor to its DNA binding sequence in order to regulate physiological differentiation process within the leukemic cells, as a first-in-class differentiation approach in cancer therapy [182,183,184]. 

### 5.2. Targeting a Pocket in the DNA-Binding Domain

However, most oncogenic transcription factors do not have natural ligand binding pocket and alternative approaches need to be developed by taking opportunities for instance of the DNA binding dynamic which may reveal potential structural pockets that might be used to select and/or design good fitting structures. This is for example achieved using the morpholine derivatives VPC-14428 and VPC-14449 (Figure 5B), two compounds specifically designed to bind a pocket of the androgen receptor (AR) DNA binding domain, a new drug-target site proposed as an alternative to the androgen binding pocket [185]. VPC-14428 and VPC-14449 induce AR inhibition by blocking its DNA binding propensity and its subsequent transcriptional activity of both AR full-length and splice variant forms (lacking the ligand binding domain) [186].

Besides protein/protein interaction inhibition, the anti-oncogene transcription factor p53 could also be targeted by direct binding. Several drugs were identified as p53 interacting compounds, either on the wild-type or the mutated p53 proteins (Figure 5C). This is the case of ellipticine binding to wild-type p53 (WT-p53) resulting in an increase in its nuclear localization and subsequent p21 promoter transactivation, as well as to the oncogenic mutated p53 (mut-p53) present in multiple cancers and hemopathies [187,188,189] to restore its normal conformation and activity [190]. Mut-p53 reactivation to restore normal p53 function is also obtained upon treatment with CP-31398, Reactivation of p53 and Induction of Tumor Cell Apoptosis (RITA), STIMA-1 or PRIMA-1 as reversible or covalent binders of mut-p53 (Figure 5) [191,192]. For instance, PRIMA-1 has potent anti-tumor activity in mut-p53 positive pancreatic cancer cells PANC1 and BxPC3 and enhances chemosensitivity toward various chemotherapeutic drugs (cisplatin, gemcitabine or doxorubicin) [193]. PRIMA-1 derivatives were also developed such as APR-246/PRIMA-1Met which induces apoptosis in mut-p53 expressing small cell lung carcinoma [194] and synergizes with other therapeutic approaches such as alkylating drugs, PARP or kinases inhibitors [195,196,197]. More recently, the 2-sulfonylpyrimidines PK11007 was identified as a new p53 inhibitor through stabilization of p53 via thiol alkylation of two surface-exposed cysteines. PK11007 bonding does not alter p53 DNA binding activity and induces the reactivation of p21 and PUMA proteins expression and other genes associated with cell death and apoptosis as evidenced using RNAseq on breast cancer triple-negative cells [198,199].

Another example is the targeting of STAT (signal transducer and activator of transcription) transcription factor family members by the 4-[(3*E*)-3-[(4-nitrophenyl)-methylidene]-2-oxo-5-phenylpyrrol-1-yl] benzoic acid (InS3-54, Figure 5D). Indeed, InS3-54 directly blocks the interaction between STAT3 and its target DNA sequence as evidenced using EMSA but does not affect STAT1/DNA binding. InS3-54 interacts non-covalently with STAT3 DNA binding domain, without affecting STAT3 homodimerization and phosphorylation [200]. However, binding affinity and STAT3/DNA binding inhibition were only obtained at high concentrations (IC_50_ ≈ 30 µM) Consequently, InS3-54 treatment of human breast and lung cancer cell lines results in apoptosis but also in reduced cell migration and invasion, in correlation with reduced expression of the STAT3 downstream target MMP-9. InS3-54 also inhibits STAT3 binding to cyclinD1 (CCND1) promoters in ChIP experiments and reduces tumor growth and metastasis [201]. Similar observations were made with InS3-54A18 derivative that inhibits STAT-3 controlled genes expressed upon IL-6 stimulation such as survivin gene [201]. By contrast, STAT3 homo-dimerization could be inhibited by irreversible bonding of C48 (NSC-368262, Figure 5D) to Cys468 residue of STAT3 within the DNA binding domain, leading to direct inhibition of DNA interaction. C48 also inhibits the DNA binding activity of STAT3/STAT1 heterodimer but not STAT1 homo-dimer [202]. This activity on different protein complexes (for instance here STAT3/STAT1 and STAT3/STAT3) may be a disadvantage if one complex is not associated with the oncogenenic process but to physiological processes, or an advantage if all complexes are associated with the oncogenic pathways (for instance in the case of an overlapping of transcription factor activities). 

Another STAT family member, STAT5, is also inhibited through direct interaction of an aptamer peptide mimicking its DNA binding-domain at the dimer interface that links the DNA helix. This binding results in the inhibition of the protein/DNA complex formation and inhibits subsequent target gene expression such as cyclinD1 and proliferation in prostate (PC3) and breast (A431) tumor models [203] as well as in chronic myeloid leukemia (K562) models [204]. 

Other compounds were developed to target transcription factors, but it is not yet clear whether they directly inhibit the transcription factor or whether they affect some unclear or yet unknown protein/protein interactions. Among them is GANT61 (2′-[[dihydro-2-(4-pyridinyl)-1,3(2*H*,4*H*)-pyrimidinediyl]-bis(methylene)]-bis[*N*,*N*-dimethylbenzenamine, Figure 5E) targeting GLI1 by interaction with the zinc finger 2 and 3 of GLI1 and GLI2 DNA binding domain but not on other zinc finger family members [205]. GANT61 inhibits GLI1/2-controlled luciferase expression at high doses (IC_50_ ≈ 10 µM), abolishes GLI1-regulated genes [206] and subsequent megakaryocytic differentiation [207]. GANT61 also induces apoptosis in many cancer and leukemia models such as gastric cancer [208], Ewing sarcoma [209], biliary tract cancer [210], lung cancer [211], breast cancer [212,213], prostate carcinoma [214,215] and adult T-cell leukemia or acute myeloid leukemia [216,217]. GANT61 induces autophagy in pancreatic ductal adenocarcinoma cells and prevents cellular migration in osteosarcoma metastasis [218] and ovarian and breast cancer invasion [212,219]. GANT61 also sensitizes cancer cells to radiation in prostate cancer model [220], to alkylating drugs such as temozolomide in glioma cells [221] or cisplatin in large cell neuroendocrine carcinoma of the lung [222] and to FLT-3 kinase inhibitor in acute myeloid leukemia [217]. 

BRD32048 (Figure 5F) is another tri-cyclic compound that targets the DNA binding domain of a transcription factor. Indeed BRD32048 directly binds ETV1, a member of the ETS transcription factor family translocated in prostate cancer and Ewing sarcoma, and inhibits its transcriptional activity on MMP1 promoter. In agreement with MMP1 reduced expression, BRD32048 inhibition of ETV1 reduces cancer cell invasion and proliferation in both LNCaP (prostatic) and 501mel (melanoma) ETV1-dependent cell lines, but not in PC3 as an ETV1-independent prostatic cell line [223]. 

More recently, virtual screening of compounds that could interact with Pax2 DNA binding domain found candidates from which was obtained EG1 (Figure 5G). EG1 binds the pared domain of Pax2, resulting in an inhibition of Pax2/DNA binding and subsequent target genes expression control, as well as reduced cell survival in renal cell (RCC11) and ovarian (SKOV-3) carcinoma models [224]. Similar approach was used to target the heat-shock transcription factor HSF1 and identified compound I_HSF1_115 (Figure 5H) for its binding in a putative pocket of the HSF1 DNA binding domain. However, I_HSF1_115 does not abolish HSF1 binding to the DNA but inhibits its hetero-dimerization with ATF1 [225]. Very recently, the FOXM1/DNA binding surface was analyzed by molecular dynamic simulations and identified a binding pocket [226] that is recognized by FDI-6 (NCGC00099374) (Figure 5I) which was previously identified, together with FDI-10 and FDI-11 compounds (Figure 5I), as able to destabilize FOXM1/DNA binding, downregulate the expression and block FOXM1 occupancy on the promoter of CDKN3, AURKA and NEK2 FOXM1-controlled genes [227,228]. Altogether, direct targeting of transcription factor DNA binding through the interaction with the DNA binding domain itself is an emerging approach with promising results and an approach that would certainly need to be further developed in the future.

## 6. Targeting Transcription Factor at the Protein/DNA Interaction Level

DNA is by itself an anti-cancer target for conventional therapies still used since the advent of anti-cancer chemotherapies near 70 years ago from the first use of 6-mercaptopurine as a first DNA alkylating drug to treat leukemia and lymphoma in clinic. After alkylating drugs, non-covalent drugs such as DNA intercalators or major/minor groove DNA ligands were developed. In the 80’s, the idea of targeting DNA at specific sequences emerged, leading to the development of many sequence-specific DNA binding compounds in order to develop a “targeted chemotherapy” against DNA. Such strategy is based on the consequences of sequence-specific targeting of the DNA to interfere with DNA binding proteins such as transcription factors associated with oncogenic processes leading to proliferation or differentiation blockade, as evidenced above in the introduction section showing that ~15% of the list of the 1988 defined transcription factors are oncogenic proteins (Table 1). 

Drug interaction with DNA could occur through different mode of binding to the DNA helix: alkylation leading to covalent bonding, intercalation between adjacent base pairs, binding to the major or minor grooves of the DNA helix. Examples of each of those sub-families of DNA interacting drugs are presented below.

### 6.1. DNA Alkylating Drugs for Transcription Factor DNA Binding Modulation

**Figure 6:**
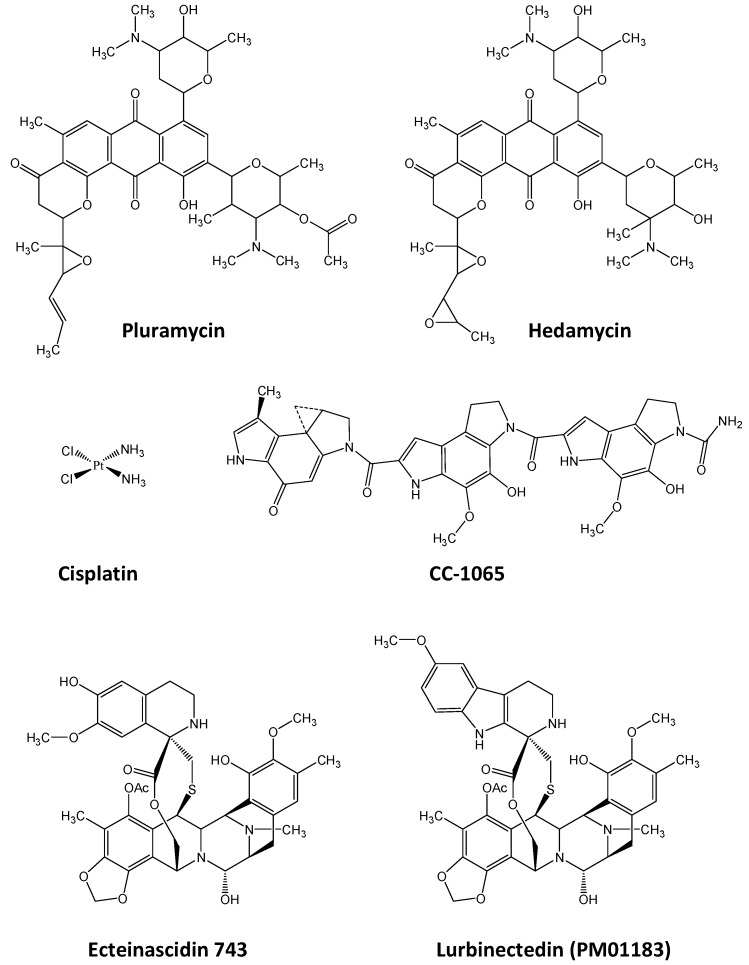
DNA alkylating drugs as inhibitors of transcription factors/DNA binding.

The first DNA alkylating drugs identified to interfere with transcription factor/DNA recognition was pluramycin [229] (Figure 6). Pluramycin is a guanine alkylating drug that forms a covalent bond with the N7 atom orientated in the major groove of the DNA helix preferentially at 5′-CGT and 5′-CGG sequences, resulting in a strong DNA unwinding and bending by an angle of 180° [230] that, when located in the close vicinity of the pluramycin alkylation site, facilitates the binding of the TATA-box binding protein (TBP) to the TATA-box of gene promoters within the TFIID basal transcription factor complex. TBP is indeed a general marker of cell proliferation and is often over-expressed in cancer cells relatively to non-cancerous cells and is associated with poor prognosis. TBP binding to pluramycin-alkylated TATA-box containing oligonucleotide has much better affinity than to unalkylated TATA-box containing oligonucleotide. As a consequence, pluramycin traps the TBP protein to its consensus binding site to form a TBP-DNA-pluramycin ternary complex [231]. Moreover, TBP binding to TATA-box results in a distorted DNA that facilitates pluramycin alkylation (Henderson 1996). This trapping of TBP by pluramycin contrasts with the effect of the pluramycin derivative hedamycin (Figure 6) that inhibits TBP/DNA binding [232]. Hedamycin is another potent inhibitor of TBP transcription factor DNA binding [233] but also seems to interact with NF-E2/AP-1 motif even if their transcrioption factor DNA inhibitory effect was not evidenced [234]. 

The second well known DNA-alkylating drug family that interferes with transcription factor/DNA binding is that of the platinated agents. Indeed, cisplatin (*cis*-diaminedichloridoplatinum(II), Figure 6), discovered more than 50 years ago and used in clinic since 40 years is a bis-alkylating drug that leads to intra- or inter-strand DNA crosslinks or to monovalent DNA adducts. Both cisplatin adducts result from covalent bonding to the N7 position of guanines in the major groove with preferential sequences for 5′-GpG intra-strand cross-links resulting in a DNA bending of 55–78° toward the major groove that destabilizes the Watson-Crick base pairing resulting in local denaturation of the DNA helix [235,236] that could span up to 7 bp for some intra-strand crosslinks [237]. Consequently, cisplatin/DNA adducts trap HMG proteins through the insertion of the Phe37 residue of the HMG DNA binding domain (HMG-box) in the bent area formed upon the two vicinal platinated guanines with the 5′-GpG dinucleotide that perfectly fits with the L-shaped structure of the HMG-box and reduces the “cost” of DNA bending for HMG-box [238] but also through interaction of Lys7 residue with cytosine residue in close vicinity of the cisplatin adduct on guanines [239]. Cellular experiments highlight that both the oncogenic HMG-B1 and HMGB-B2 [240] participate in platinated-agent-induced cytotoxicity [241]. This platinated distorted DNA is also a good substrate for other proteins, such as HMG-B4 [242] and other transcription factors containing HMG-boxes such as SRY, LEF-1 or TOX4 [243,244,245] that are associated with cancer stemness [246], and targets for cancer treatment [247]. TBP also shows preferred binding to platinated DNA relatively to unmodified DNA with a 175-fold increase in the binding affinity and a decrease of >30-fold of the TBP/DNA dissociation constant [248]. Sp1 and Sp3 transcription factor binding to their cognate sequence is also increased upon cisplatin treatment of the DNA as evidenced using gel shift assays [249]. By contrast, platinated adducts on κB consensus DNA binding sites distort DNA helix and consequently inhibit NFκB transcription factor binding to κB-DNA [250]. Similarly, DNA platination abolishes p53 and p73 binding to DNA on p21 and MDM2 promoter sequences [251,252]. Beside platinated compounds, other metal-associated molecules may also perturb transcription factor/DNA binding, as for chromium-derivatives that inhibit p300/CBP protein binding to DNA [253] as well as CTCF and AP1 binding to their consensus binding sequences on the DNA as recently evidenced in hexavalent chromium-derivatives treated cells [254]. 

CC-1065 (Figure 6) is an antibiotic minor groove alkylating drug in pre-clinical development that bonds to the N-3 position of guanines to bend the DNA helix and that interferes with transcription factor/DNA binding: CC-1065 inhibits TBP/DNA interaction [233] but facilitates the binding of SP1 transcription factor to DNA [231,255].

The tetrahydroquinoline alkaloid ecteinascidin-743 (ET743, Trabectedin, Yondelis^®^) is another minor groove alkylating drug that interferes with transcription factor binding to DNA. ET743 (Figure 6) reacts with the exocyclic amino group of guanine to form a DNA adduct orientated toward the minor groove which increases the size of the major groove on the opposite strand, in a sequence-dependent manner [256,257,258]. As a consequence, ET743 modifies DNA conformation and inhibits several transcription factors DNA binding like TBP, E2F and NF-Y, resulting in the inhibition of the expression of the multi-drugs exclusion pump MDR1, associated with chemoresistance [259,260]. ET743 also induces the displacement of HMGA protein from the ATM promoter [261]. ET743 also inhibits FUS-CHOP transcription factor binding to the promoters of different genes among CHOP, pentraxin 3 and fibronectin 1 to restore adipogenic differentiation in myxoid liposarcoma, a cancer against which ET743 entered into phase I/II clinical trials [262,263,264,265]. Moreover, ET743 and its derivative lurbinectedin (PM01183) inhibit the DNA binding activity of the fusion transcription factor EWS-FLI1 responsible for pediatric Ewing sarcomas. This inhibition results in a change in EWS-FLI1 nuclear and nucleolar distribution as well as a decrease in the promoter activity of NR0B1as, an EWS-FLI1 controlled gene, but also the expression of other key controlled genes such as EZH2, ID2 or KMO [266,267]. More recently, ET743 also alters DNA binding of another fused transcription factor protein: EWS-WT1 that originates from t(11;22)(p13;q12) and is responsible for desmoplastic small round cell tumors. Indeed, EWS-WT1 interaction with EGFR promoter is reduced upon ET743 treatment in JN-DSRCT-1 cells as evidenced using ChIP experiments [268]. 

All of these examples rely on potent or potential anti-cancer drugs that seem to possess two key mechanisms of action: DNA alkylation and transcription factor/DNA binding modulation. The consequences of DNA alkylation are, first and foremost, the induction of a maximum of DNA damages leading to cell death in cancer cells or any other cycling cells, as treated cells do not have time to correctly repair a multitude of simple DNA damages (adducts) and thus accumulate poorly manageable DNA damages such as double strand breaks. Alteration of transcription factor activities may appear marginal from comparison with the impact of DNA alkylation process. However, this additional function against a transcription factor which would be associated with tumor development (as presented above for ET743 on EWS-FLI1) may lead to a better therapeutic index because of different consequences between treated cancerous and normal tissues.

### 6.2. DNA Intercalating Drugs for Transcription Factor DNA Binding Modulation

**Figure 7:**
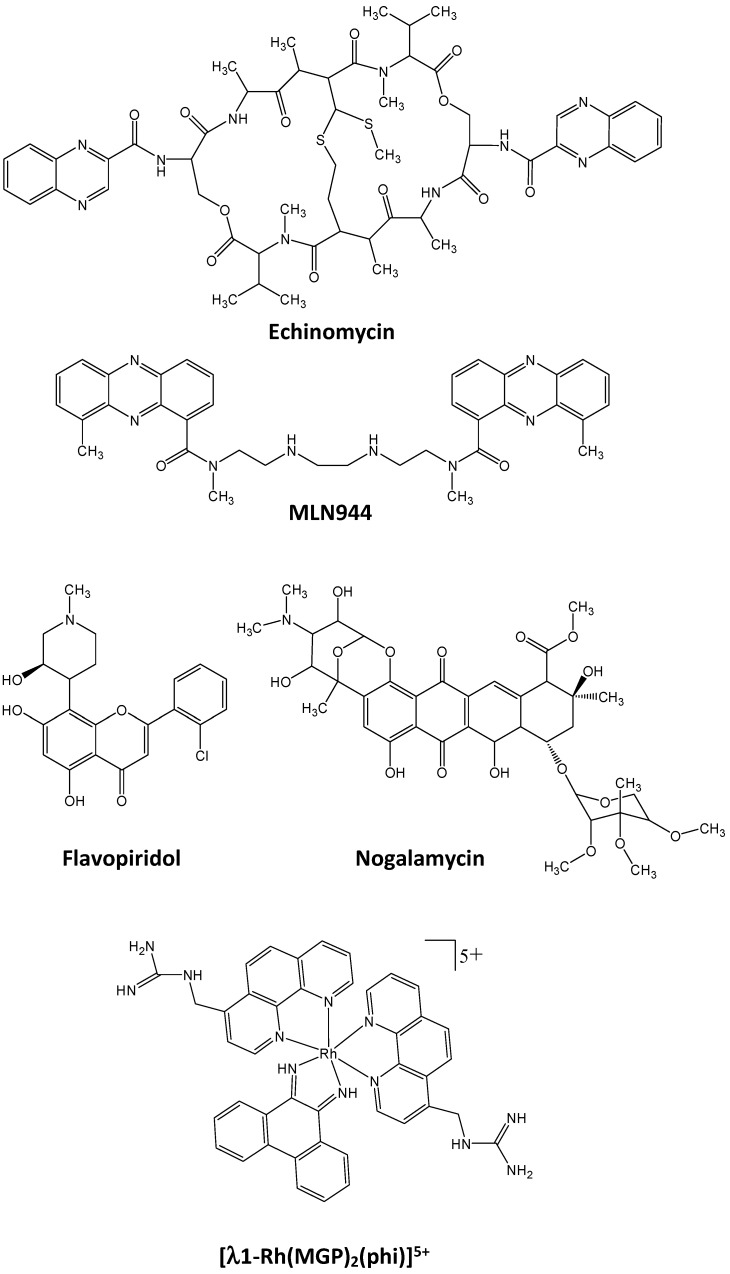
DNA intercalating drugs as inhibitors of transcription factors/DNA binding.

The intercalation process of aromatic chromophores between two consecutive base pairs of the DNA induces specific DNA constraints to the DNA helix, associating an increase in the DNA length from 3.4 Å to around 6.8 Å, and an unwinding of the DNA helicity associated with increased DNA constraints [269]. For instance, the anthracycline derivative daunorubicin unwinds the DNA by an angle of 15° [270], ethidium bromide by 17° or 26° depending on DNA sequences [271,272] and actinomycin D induces a 28° rotation [273]. Both DNA elongation and unwinding may by themselves affect usual deep contacts of a transcription factor with DNA. The alteration is moreover reinforced by the presence of portions of the molecule that may protrude of one and/or the other side of the “stairs” formed by the successive base pairs of the DNA. This is particularly important for large compounds that intercalate in the perpendicular orientation and for which portions of the molecule partially fill the major and/or the minor grooves of the DNA helix, thus limiting access to DNA of amino acid residues of the transcription factors DNA binding domains. Moreover, some intercalating drugs may have two intercalating domains that are positioned between two series of adjacent base pairs. Due to the length and rigidity of the molecule linker portion that associates with the two intercalation motifs, bis-intercalation may also distort the DNA axis toward the major or the minor groove as do alkylating drugs. For instance, the peptide antibiotic echinomycin (NSC-13502, Figure 7) unwinds the DNA helix by an angle of 48° together with a DNA bending [274]. At last, DNA structure modulation upon drug binding is also impacted by the natural DNA breathing associated with the formation of Hoogsteen base pairing in which the purine bases flips from the anti to the syn orientation, and with a change in the number (two at maximum) and distances of the hydrogen bonds between AT and GC base pairs thus reducing the minor groove width from around 10.5 to 8.5 Å [275]. This was recently well demonstrated using NMR and molecular dynamics on echinomycin binding to DNA [276]. In terms of transcription factor inhibition, echinomycin was identified as a HIF1α and HIF1β/DNA binding inhibitor as evidenced using EMSA, luciferase activity and ChIP experiments on HIF-1 response elements (HRE) of the vascular endothelial growth factor promoter (VEGF, associated with tumor vascularization) [277]. Echinomycin also induces HIF1/DNA binding inhibition to control REDD1 [278], endothelin-2 [279], GLUT3 [280], GLUT1, BCL2 and NOTCH1 in leukemic cells [281] as well as the human growth hormone promoter [282]. Consequently, it inhibits cell proliferation [281] and induces apoptotic cell death [283]. The echinomycin biosynthetic precursor triostin A and its derivatives are also potent inhibitors of HIF1 effective on hypoxia model and induce cell death in MCF7 cancer model [284].

MLN944 (XR5944, Figure 7) is a bis(phenazine-1-carboxamide) compound, another bis-intercalator that binds to DNA through its two phenazine rings intercalating (arrows) in the 5′-AT-GC-AT-3′ palindromic sequence and the amino-carboxamide linker lying along the major groove of the 5′-GC-3′ portion of DNA to induce a right-handed twist of the DNA helix with unwinding of 48°. As a consequence, MLN944 inhibits c-JUN/DNA binding on the AP-1 5′-aTGAGTCA-3′ sequence [285] but also the estrogen receptor alpha ERα/DNA binding on the palindromic estrogen response element (ERE) 5′-AGGTCAnnnTGACCT-3′ [286,287].

Mono-intercalating drugs may also block transcription factor/DNA binding as demonstrated with flavopiridol (alvocidib, Figure 7). Besides its activity of cyclin-dependent kinases (CDKs) inhibitor, flavopiridol interacts with DNA with strong affinity and inhibits STAT3/DNA binding [288]. Flavopiridol lowers STAT3-directed transcription on STAT3-driven promoters in luciferase assays and also down-regulate the expression of STAT3 controlled genes such as MCL1 [289]. Because STAT3 is commonly overexpressed in AML, flavopiridol remains of particular interest against this hematological disorder [290]. Some metallo-intercalators associating ruthenium or platinum atom to stabilize rings in a planar configuration were also evidenced as inhibitors of protein/DNA binding like for example, [Ru(phen)_2_(dppz)]^2+^ against the interaction of PUrine-rich box-1 (PU.1)/SFFV Proviral Integration Site-1 (SPI1), an ETS-family member transcription factor, to its minimal cognate ETS-family-core binding site 5′-GGAA/T-3′ [291], or ethaRAPTA against the DNA repair protein BRCA1 also associated with transcription control [292]. 

As another example, the anthracycline nogalamycin (Figure 7) inhibits EGR1 and AP1 through intercalation between G/C-base pairs in a perpendicular manner, presenting part of the molecule in both grooves [293,294]. The intercalation is associated with a 35° helical twist [295] that facilitates the inhibition of TBP binding on the TATA-box in close vicinity of a GC-rich site that nogalamycin binds and bends. However, as an anthracycline, nogalamycine is also a topoisomerase inhibitor and therefore its anti-tumor activity may also be exerted through a general DNA topoisomerase inhibition process. 

Finally, several metal-coupled compounds intercalate in the DNA helix with some sequence selectivity due to major groove contacts to selectively inhibit transcription factor DNA binding, as demonstrated with the lambda-1-Rh(4-guanidylmethyl-1,10-phenanthroline)_2_ (9,10-phenanthrene- quinone diimine)^5+^(Lambda-1-Rh(MGP)_2_phi^5+^ (Figure 7) that binds specifically the AP-1 recognition element 5′-CATATG-3′ in the major groove through its guanidinium moiety that bonds DNA toward the major groove with a 70° unwinding of the DNA helix. As a consequence, lambda-1-Rh(MGP)_2_phi^5+^ inhibits AP-1 transcription factor binding to DNA, even if only at sub-micromolar concentrations [296]. 

As for alkylating drugs associated with the modulation of transcription factor activites presenting double mode of activities, the intercalation of molecules between stacked bases of the DNA helix have per se cellular consequences that are associated with anti-cancer activities and induce death of cycling cells that are responsible for the drugs toxicity in treated patients and some limitation of their use in the course of cancer treatment. It could be direct effect on the replication machinery or through DNA topoisomerase inhibition. The intercalating drugs described above for modulating transcription factor/DNA binding, but nogalamycin, are not topoisomerases inhibitors (Echinomycine, MLN944, flavopiridol) or not known as such but may have alternative mode of action typical of DNA intercalation (as for instance on DNA polymerases) or is also cyclin-dependant kinases inhibitor (flavopiridol). Multiple mode of action make difficult to address the anti-tumor activity of a compound to one or another of its target.

### 6.3. Major Groove DNA Binding Drugs for Transcription Factor DNA Binding Modulation

Based on the asymmetry of the DNA helix, the left and right side of the helix are not equal: a shallow-wide major groove and a deep-narrow minor groove are formed and represent two different opportunities to bind the DNA helix along the axis. The major groove has a bigger size (11.6 Å) and is a good template for protein binding through the helices from different families of DNA binding domains but a poor one for small molecular weight compounds that better fit in the minor groove (6.0 Å). Therefore, transcription factor inhibitors that may target the DNA major groove in a sequence-selective manner might easily compete for transcription factor binding to their cognate sequences. 

**Figure 8:**
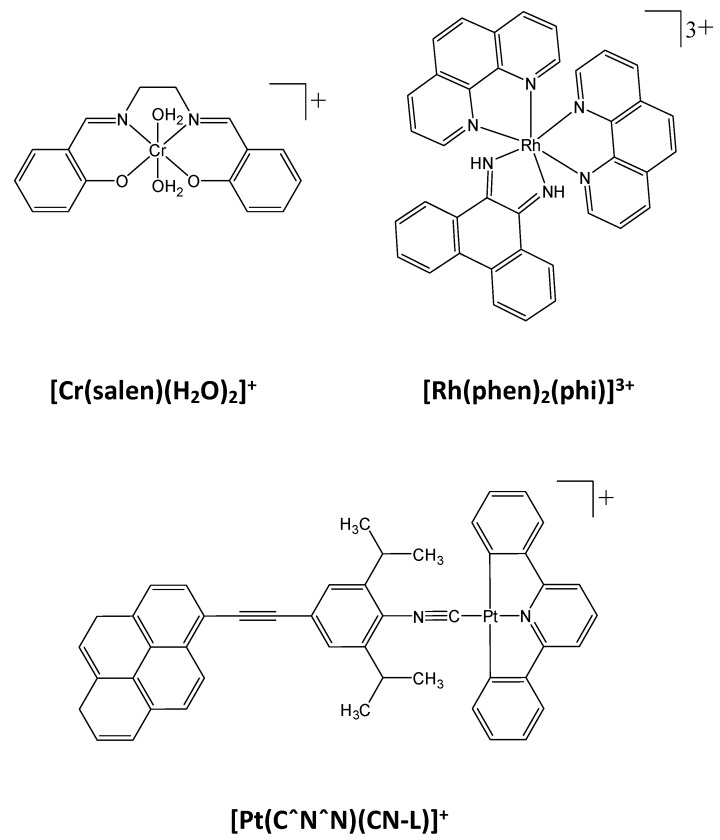
Major groove DNA binding drugs as inhibitors of transcription factors/DNA binding.

The size of the major groove is compatible with that of another strand of DNA making contacts with, Hoogsteen bases and therefore forming triple-stranded DNA helix [297,298]. The additional DNA sequences called triplex-forming oligonucleotides (TFOs) would consequently interfere with transcription factor DNA binding as already discussed in several reviews and will not be further presented here [299], we will focus on the few number of small compounds that interact with the major groove. Among them are essentially metal-coupled compounds that associate chromium, ruthenium, platinum ions in a polycyclic structure (Figure 8). For instance, [Cr(salen)(H_2_O)_2_]^+^ is able to disrupt SP1 and TFIID DNA binding through its interaction with the major groove [300] and the ruthenium complex [Rh(1,10-phenanthroline)_2_(9,10-phenanthrenequinone diimine)]^3+^([Rh(phen)_2_(phi)], Figure 8) inhibits RNA transcription in vitro [301]. In a similar manner, some platinum complexes such as the square planar pyrene-coupled platinum(II) complex [Pt(CˆNˆN)(C≡N-L)]^+^ (where L = 4-(3,5-diisopropylphenylethynyl)pyrene, Figure 8) binds into the major groove of the DNA and blocks the cAMP response element binding protein (CREB) binding to its response element CRE as evidenced by EMSA [302]. Such inhibition could only be reached at high concentrations with IC_50_ ≈ 40 μM in order to inhibit CREB binding to CRE-containing DNA but is however specific as demonstrated with other transcription factors (JUN/FOS, NFκB) which interaction with their respective cognate sequences could not at all be disrupted at the highest evaluated concentration of drug (80 µM) [303]. Such high concentration of drug required for abolishing CREB/CRE binding might strongly compromise their use in clinic. 

### 6.4. Minor Groove DNA Binding Drugs for Transcription Factor DNA Binding Modulation

Only a few number of low molecular weight groove binders interacts with the DNA major groove, where most of the proteins bind; the vast majority of molecules have easier propensities to fit in the DNA minor groove due to higher number of van der Waals contacts in this narrower groove, relatively to the major groove. 

**Figure 9:**
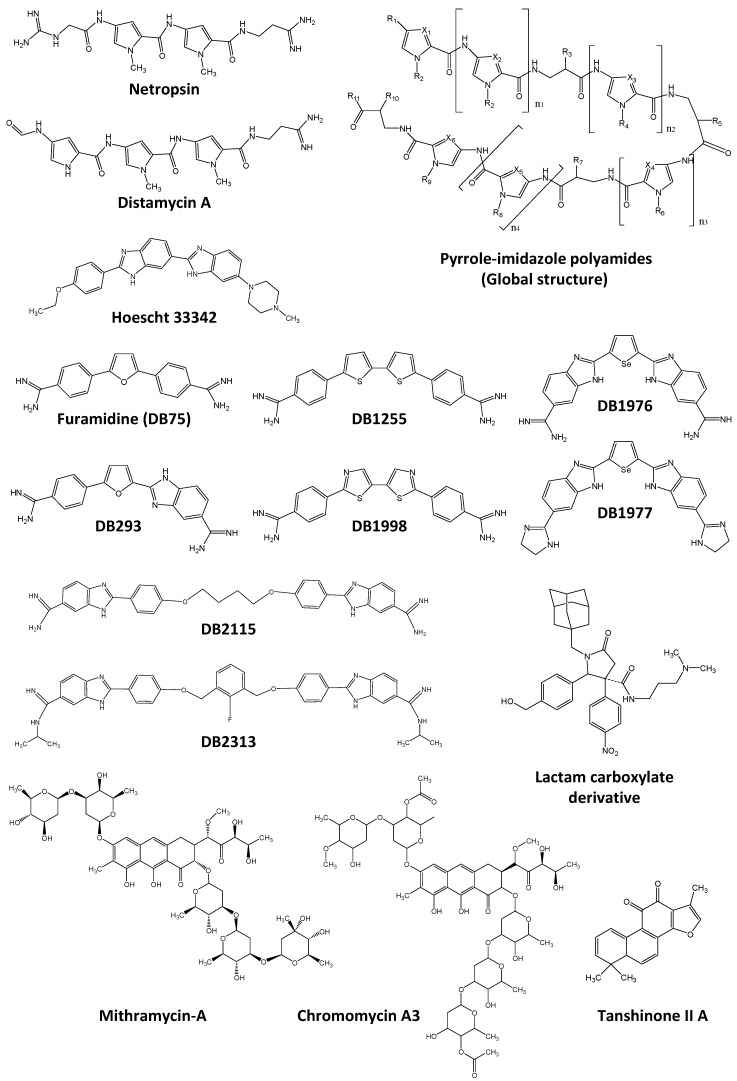
Minor groove DNA binding drugs as inhibitors of transcription factors/DNA binding.

The first family of minor groove DNA binding compounds known since ~40 years to inhibit transcription factors/DNA binding is the one including netropsin, distamycin and Hoescht 33258 (Figure 9) containing a succession of 3 to 4 rings, organized as a crescent to fit intimately with the natural curvature of the minor groove over 3–4 bp, and positive charges at both extremities which make direct or water-mediated contacts with the DNA. All three compounds recognize stretches of AT-rich base pairs in the minor groove of the DNA helix in a relatively unspecific manner [304,305]. 

Netropsin binds as a monomer in the minor groove of 3–4 successive A or T bases and inhibits AT-rich binding transcription factors such as TBP and its co-factor TFIIA [233] or HMGA1 that failed to interact with the NOS2 promoter [306]. 

Distamycin A binds as a monomer in the minor groove of four successive A-T base pairs such as on 5′-ATTA-3′ sequence. But distamycin A also binds as a dimer on 5′-AAGTT-3′ sites, for instance [307]. The dimeric binding of distamycin A occurs in a cooperative binding mode on many AT-rich sequences with some exception such as alternative succession of A or T bases as observed in 5′-ATAT-3′ site which is recognized as dimer in an anti-cooperative manner. Dimer of distamycin A fitted in the minor groove results in an increase of the size of the minor groove associated with a decrease of the size of the major groove on the opposite side, as well as a large bending of the DNA axis toward the major groove [303]. As a consequence of these different modes of binding, distamycin A inhibits DNA interaction of different transcription factors such as OTF-1 and NFE-1 [308], OCT-1 [309], TBP [233], the Epstein-Barr virus nuclear antigen 1 [310], E2F1 [311], NFκB, HMGA1 [312,313] and PU.1/SPI1 [314].

Hoechst 33342 is a bisbenzimidazole derivative that binds to AT-rich DNA minor grooves as a momoner, as a dimer, and probably also as a tetramer. However, Hoechst 33342 also intercalates in GC-rich sites at higher Hoechst 33342 concentration [315]. Consequently, Hoechst 33342 inhibits the interaction of TBP to the TATA-box, of ELK-1/SRF complex to the serum response element (SRE) [316] and of GFI-1 to an AT-rich site on the survivin promoter [317]. Microgonotropens (FMGTs) are Hoechst derivatives that also inhibit transcription factor DNA binding such as E2F1 binding on the dihydrofolate reductase promoter [318] or the ELK-1/SRF complex binding to the SRE sequence present on c-Fos promoter [319]. 

Destabilization of transcription factor/DNA complexes by netropsin, distamycinA and Hoechst 33342 was not enough selective to one precise transcription factor or transcription factor family. But due to their interesting DNA binding properties, those three compounds were the first building blocks used to develop other sequence-selective minor groove DNA binders designed to increase the size and/or the specificity of the interacting DNA sequences and therefore to increase the specificity of the inhibitory effect of those drugs on transcription factor/DNA inhibition. Two main series were developed: “polyamides” using a succession of several well-defined building blocks and “heterocyclic diamidines” using a fewer number of building blocks but presenting a bigger variety of structures.

Pyrrole-imidazole polyamides (see global structure in Figure 9) was the first developed approach and is based on the successive addition of a few number of well-defined building blocks, each being more or less specific for a defined base pair. For instance, imidazole-pyrrole successive rings are proposed to bind to 5′-GC-3′ while pyrrole-imidazole would rather interact with 5′-CG-3′ steps and pyrrole-hydroxypyrrole and hydroxypyrrole-pyrrole successions would recognize 5′-AT-3′ and 5′-TA-3′ base pairs, respectively. Additional building blocks include for instance γ-turn, β-alanine, α-methoxy-β-alanine, methyl-hydroxypyrrole or *N*-methylpyrazole, α- or β-hydroxyl-γ-aminobutyric acids and imidazopyridine (for reviews see Dervan 2005; Kawamoto 2018) [320,321]. Such diversity of molecules offers a large variety of transcription factor/DNA binding inhibitors for anti-tumor activities [322]. Exemples are presented in Table 2.

Most of those polyamides strongly interact with the DNA with binding affinities often at nM range but however often require concentrations at µM range to interfere with transcription factors binding to their cognate sequences, when there are not impeded for further development due to poor cell penetration due to the size of those big molecules.

The second series of molecules used to develop inhibitors of transcription factor/DNA binding are heterocyclic diamidines deriving from both distamycin and pentamidine. Transcription factor inhibition for cancer treatment was not the first goal in the development of this series of compounds, but anti-parasitic and anti-microbial activities, such as against *Trypanosoma cruzi*, *Leishmania amazonensis* or *Pneumocystis carinii* among others infectious diseases [356]. It is against those pathologies that the diphenyl-furan-diamidine DB75 (Furamidine, Figure 9) and its pro-drug DB289 (para-furamidine) were developed [357].

DB75 binds as a monomer in the minor groove at stretches of four AT base pairs. Changing one of the two phenyl rings of DB75 by a benzimidazole moiety modifies the sequence-specificity and mode of binding: such DB293 molecule (Figure 9) now also binds as head-to-tail stacked dimers in the minor groove of 5′-ATGA sequence, leading to an increase in the size of minor groove [358] and was found to inhibit PIT-1 and BRN-3 transcription factors, two POU-family transcription factors [359]. If DB293 compete for PIT-1 and BRN-3 binding to their cognate sequences, IRF-1 transcription factor that also binds to a consensus site containing both an ATGA and an AT-rich site was not inhibited by DB293. Such difference was associated with the binding of DB293 as a dimer on the 5′-ATGA-3′ portion on the cognate sequence of PIT-1 and BRN-3 but as a monomer on the AT-rich portion of the IRF-1 consensus site as demonstrated using DNase1-footprinting experiments. Surface plasmon resonance (SPR) confirmed sequence specificity, 2:1 drug:DNA stoichiometry and evidenced a strong cooperative dimer binding on PIT-1 and BRN-3 cognate sites but evidenced non-cooperative monomer binding on IRF-1 binding site, in agreement with AT-rich, but not 5′-ATGA-3′, recognition in the context of IRF-1 DNA binding site. 

The second compound from this series that was identified as a transcription factor inhibitor is the tetracyclic symmetrical diamidinophenyl-dithiophene DB1255 on the ETS transcription factor family member ERG (Figure 9). ERG is an oncogene that is over-expressed or translocated in cancer: ERG is indeed fused to TMPRSS2 promoter region in >50% of prostate cancer [360], to EWS in 10–15% of Ewing sarcoma [361] and to FUS/TLS or ELF4 in different subtypes of leukemia [362,363] and overexpressed in leukemia where it is associated with poor prognosis [364]. DB1255 inhibits ERG/DNA binding in ELISA-derived protein/DNA binding inhibition assays (EPDBi) and EMSA, associated with the interaction of DB1255 with the 3′-end portion of the ERG cognate sequence as identified using DNase I footprinting [365]. Indeed, DB1255 recognizes at nM range the 5′-**AAGT**T-3′ site that is present in the ERG binding site 5′-**GGAAGT**-3′ when followed by an additional T base (underlined, ETS-family common cognate site, in bold ERG cognate sequence). Such specificity for the addition of a thymine 3′ to the ERG cognate sequence may presumably limit their inhibition efficiency on ERG protein binding to all potential ERG cognate sequence (with a cytosine, an adenine or a guanine steigth at the 3′-end) but this offers the opportunity to modulate ERG inhibition to some ERG-driven genes, assuming that the crucial ERG-driven genes associated with cancer development are well characterize and are controlled through a ERG cognate sequence followed by a thymine in its 3′-end. DB1255 binds as a dimer in the DNA groove as evidenced using circular dichroism and SPR [366]. Modification of the global planarity of DB1255 by changing dithiophenes in difuran (DB914) or diselenophene (DB1282) rings enlarged the thickness of the molecule over the natural width of the minor groove of the DNA helix and thus abolished the DNA binding properties of such derivatives and subsequently their transcription factor inhibitory effect. Similar results were obtained when the longitudinal axis angle measured from one amidine to the other was closer or wider as such modifications was assumed to prevent proper deep binding of both two amidines groups with the minor groove of the DNA. This is achieved by changing the dithiophenes in DB1255 into a difuran (DB914), diselonephene (DB1282) or changing diphenyl in DB1255 to dibenzimidazole rings (DB1974/DB1975). Modification of dithiophene in DB1255 to dithiazole rings in DB1998 derivative (Figure 9) was the only modification in this series that did not abolish the inhibitory affect on ERG/DNA binding, but this inhibition was less efficient than using DB1255 (IC_50_ ≈ 1 μM) which was preferred for cellular investigations. At the cellular level, DB1255 altered ERG-controlled transcription on artificial promoter and on osteopontin promoter, an ERG-driven promoter associated with prostate cancer [367]. Finally, both DB1976, DB1977, DB2115 and DB2313 (Figure 9) evidenced PU.1/SPI1 DNA binding inhibition at 2.5–5 µM range through interaction with a DNA interaction affinity around 1–10 nM on the ERG-binding site present in the λB motif of the murine Igλ2-4 enhancer that was used as a model sequence [368,369]. PU.1 is another ETS family member, sharing with ERG the common ETS-minimal binding site 5′-GGA(A/T)-3′ but having strong preference for an AT-rich track 5′ to the minimal ETS binding site [370]. PU.1 is well described as an oncogene in erythroid leukemia [371] but its oncogenic role in the genesis and prognosis of acute myeloid leukemia is still controversial since the decrease of PU.1 expression as well as heterozygous deletion in patients induces AML [372,373,374]. However, decrease in PU.1 expression was associated with a decrease in AML cell proliferation and DB2313 evidenced cellular anti-proliferative activity in a human AML cell model with IC_50_ ≈ 20 μM. Moreover, DB2313-treated cells inoculated in mice result in a decrease in the leukemia burden compared to the one induced by untreated AML cells [369]. 

Other classes of molecules also bind to the minor groove and interfere with transcription factor/DNA binding. Among metal-containing DNA binders, several platinum-acridine conjugates proved to be potent minor groove binding agents together with DNA alkylation process on the N3 reactive position of adenines such as PT-ACRAMTU [PtCl(en)(ACRAMTU)](NO_3_)_2_, en = ethane-1,2-diamine, where ACRAMTU = 1-[2-(acridin-9-ylamino)ethyl]-1,3-dimethylthiourea] that evidenced TBP/DNA binding inhibition activity [375]. The lactam carboxamide derivative (Figure 9), was identified as a competitor for DNA interaction of the homeobox protein HOXA13 (IC_50_ of 6.5 μM), a transcription factor family that is up to know poorly targeted at the DNA binding level by DNA ligands [376]. The natural phytoestrogen tanshinone IIA (Figure 9) is an AT-rich site minor groove DNA binding compounds [377] that efficiently inhibits in vitro binding of AP-1 complex to DNA [378]. Tanshinone IIA inhibits the AP-1-driven COX2 expression in H22 cells treated at 10–25 µM concentrations [379]. Tanshinone IIA also interferes with RNA polymerase II associated with altered p53 responses and induction of apoptosis [380].

Today most developed compounds for transcription factor/DNA binding inhibition are mithramycins (MTMs). Indeed, even if their specificity for DNA sequences and their affinity to those sequences are not the best one from all minor groove binding compounds presented here, those compound and derivatives entered clinical trials. MTMs represent a family of natural antibiotics isolated from different Streptomyces species such as MTM-A (Figure 9) or synthetic derivatives MTM-SK, MTM-SDK [381] and DIG-MSK [382]. MTMs bind as dimers in the minor groove of GC-rich DNA sites [383,384]. 

MTMs were first as a protein/DNA inhibitor of the SP1/3 family of transcription factors [382,385] to control the expression of many genes as presented in Table 3.

Mithramycin was also evidenced as an inhibitor of the EWS-FLI1 fusion transcription factor activity but without evidencing if it comes directly from inhibition of EWS-FLI1/DNA binding or indirectly from inhibition of SP1/DNA binding [266]. Conversely, NMR evidenced a ternary complex of MTM/DNA/FLI1, where FLI1 binds in the major groove on 5′-GGAA-3′ sites and MTM binds in the minor groove, either in close vicinity or on the FLI-1 binding site [408].

Because it controls genes associated with many pathways as evidenced above, the cellular consequences of MTMs treatment are also diverse and correspond to various ways to target the different hallmarks of cancer as defined by Hanahan and Weinberg [409]: anti-proliferative effect control of transcription, induction of apoptosis, control of metabolism and anti-angiogenic activity. MTMs entered into clinical trials against solid tumors or leukemias. For instance, it entered into a phase II trial in testicular tumors and showed interesting activity against embryonal carcinoma sub-type (Kennedy 1995) and more recently against refractory Ewing sarcoma [410]. 

Finally, chromomycin A3 (Figure 9) is another antibiotic associated with transcription factor/DNA binding inhibition. This G/C-specific minor groove binding compound inhibits EGR1, AP1, ELK-1 and, to a lesser extent, TBP [233,316].

## 7. Conclusions

Cancer is a consequence of multiple deregulated processes first defined as the “Hallmarks of Cancer” by Hanahan and Weinberg two decades ago, implicating six main processes, now revised more recently to ten different processes [409,411]. Because all cancer varies in tissue origin, genetic alterations or evolution, some authors proposed specific deregulations associated with those hallmarks for a defined cancer subtype as for leukemias, colon, head and neck and prostate cancers or glioblastoma [412,413,414,415,416,417]. Many transcription factors are associated with multiple of these hallmarks of cancers and are therefore defined as oncogenes (see Table 1). Among them are NFκB, P53, MYC, HIF-1, STATs, GLI1, ERG, RUNX1, FOXO, HOXs and NRF2 [418,419,420,421,422,423,424,425,426,427], all transcription factors against which inhibitors are developed as presented along this manuscript. For a long time, transcription factors (other than nuclear receptors for which derivatives of natural ligands have be developped) were considered as undruggable targets and indirect strategies were developed in parallel to their association to cancer processes such as the epigenetic control of their expression. Knowing more precisely the mechanism of action of each transcription factor in interaction with its cognate DNA sequence or protein partners opened new opportunities to develop therapeutic approaches such as protein/protein interaction inhibitors, sequence-specific DNA ligands and more recently pocket-binding ligands evaluated in the dynamic of transcription factor/DNA or protein interaction. 

Among the various strategies presented in this review (change in transcription factor expression level, stabilization/degradation modification, modulation of transcription factor/protein or transcription factor/DNA interactions or direct binding to transcription factor), all present advantages and disadvantages that are moreover to see in light of the nature and function of each transcription factor. 

About the nature of the target transcription factor, the reactivation of mutated p53 is an interesting model to highlight how the complexity of cancer biology interferes with drug discovery. Indeed, mut-p53 cannot be considered as a unique entity since many different point mutations (single or multiple) affect this transcription factor function leading to cancer. As a matter of fact, hotspot and minor mutations could occur in the DNA binding domain of mut-p53 protein affecting only its conformation (for instance R175H, G245, R249S and G215D) or together with p53 interaction with DNA (such as R248Q, R273H, R248W, R273C and R282W) [428]. Mut-p53 reactivating drugs such as CP-31398, RITA, STIMA-1 or PRIMA-1 differently interact with the different mut-p53 proteins: CP-31398 stabilizes and changes the conformation of R273H but also R249S mut-p53 proteins; RITA-1 is active on R273H, R175H, R248W and R280K mut-p53 whereas PRIMA-1 and STIMA-1 binds only to R273H and R175H mutants [429]. Consequently, the use of such reactivating drugs would be deeply dependent on a fine characterization of p53 mutation for each patient and so would be a nice model for potential personalized medicine. 

Furthermore, transcription factors activate or repress wide repertories of downstream target genes which differ depending on cancerous or normal cell type. Identifying an oncogenic signature from normal function repertories of genes driven by this transcription factor is not always easy due to some redundancy and the difficulty to establish the threshold of expression associated with the oncogenic process. 

Moreover, for DNA ligands, DNA recognition context is important as illustrated with DB1255. Indeed, DB1255 recognizes only the ERG binding site 5′-**GGAAGT**-3′ followed by an additional T base (underlined, ETS-family common cognate site, in bold ERG cognate sequence), corresponding statistically to ¼ of the total of ERG cognate sequence on the genome. Such specificity may be limitated (considering that only a potion of ERG-driven genes could be deregulated) or may offer the opportunity to modulate more precisely ERG-driven genes assuming that they are associated with the oncogenic process. Identifying such set of deregulated genes depending not only from the presence of an ERG cognate sequence but also of the surrounding bases through bioinformatics tools would be helpful for future development of such type of compound.

In a general manner, a better knowledge of the precise modalities of transcription factor/DNA or co-factor binding would take benefit of global analysis using high throughout analysis and next-generation sequencing (transcriptome, RNase-seq, ChIP-seq, DNase-Seq, ATAC-seq, SELEX-seq and ChIA-Pet, methylome and exosome analyses, etc.) of these transcription factors and of their different co-factors in the context of each tumor cell type, particularly from comparison with normal cell context and would be helpful for future design of transcription factor inhibitors. 

Futhermore, transcription factors could interact with some epigenetic modifiers and their cooperation regulates gene expression pathways that are responsible for the cellular oncogenic phenotype [430]. A better knowledge of the setting up distorsion of transcription factors and the epigenome installed in diseases will offer hope to develop new opportunities. With this goal, we can notice the important clinical use of epigenetic therapies, either alone or in combinaison therapies for cancer treatment [431]. 

Moreover, the development of specific transcription factor inhibitors against cancer would have to take into account compensation phenomena between different transcription factors that may be very close to each other in the same family. 

Finally, the dynamics of transcription factor/DNA or co-factor interaction process have to be further elucidated to open new opportunities to develop inhibitors that would block transcription factor 3D orientation in an active or an inactive structure. 

Altogether, if the inhibition of transcription factor to treat cancer is already a part of the current anti-tumor pharmacopoeia, it would surely be improved in the future and opened to other pathologies such as genetic or inflammatory diseases, diabetes, Parkinson and Alzheimer diseases. 

## Figures and Tables

**Table 1:** List of the 294 known or candidate oncogenic transcription factors and regulators ^1^.

ABL1	CEBPA	ERCC3	HIST1H2BE	MDM4	PAX7	SMARCA4	TFPT
AFF1	CHD1	ERCC6	HIST1H2BG	MED12	PAX8	SMARCB1	THRAP3
AFF3	CHD2	ERF	HLF	MEF2B	PBX1	SMARCD1	TLX1
AFF4	CHD4	ERG	HMGA1	MEF2C	PEG3	SMARCE1	TLX3
APC	CHD5	ESPL1	HMGA2	MEN1	PER1	SMURF2	TNFAIP3
AR	CHD7	ESR1	HOXA11	MITF	PHF3	SOX2	TP53
ARID1A	CIC	ETS1	HOXA13	MKL1	PHF6	SOX5	TRIM24
ARID1B	CIITA	ETV1	HOXA7	MLLT1	PHOX2B	SOX9	TRIM33
ARID3B	CNOT3	ETV4	HOXA9	MLLT10	PLAG1	SRCAP	TRIP11
ARID5B	CREB1	ETV5	HOXC11	MLLT3	PML	SS18L1	TRPS1
ARNT	CREB3L1	ETV6	HOXC13	MLLT6	PMS1	SSB	TRRAP
ARNT2	CREBBP	EWSR1	HOXD11	MYB	PNN	SSX1	TSC22D1
ASB15	CRTC1	EYA4	HOXD13	MYBL1	POU2AF1	SSX2	TSHZ3
ASXL1	CSDE1	EZH2	ID3	MYC	POU2F2	SSX4	VHL
ATF1	CTCF	FEV	IRF2	MYCN	POU5F1	STAT3	WHSC1
ATF7IP	CTNNB1	FLI1	IRF4	MYOD1	PPARG	STAT4	WHSC1L1
ATM	DACH1	FOXA1	IRF6	NCOA1	PRDM1	STAT5B	WT1
ATRX	DACH2	FOXE1	IRF8	NCOA2	PRDM16	STAT6	WWP1
BAZ2B	DAXX	FOXL2	IRX6	NCOA4	PRDM9	SUFU	WWTR1
BCL11A	DDB2	FOXP1	JUN	NCOR1	PRRX1	SUZ12	XBP1
BCL11B	DDIT3	FOXQ1	KHDRBS2	NCOR2	PSIP1	TAF1	XPC
BCL3	DDX5	FUBP1	KHSRP	NEUROG2	RARA	TAF15	ZBTB16
BCL6	DEK	FUS	KLF2	NFE2L2	RB1	TAL1	ZBTB20
BCLAF1	DIP2C	FXR1	KLF4	NFE2L3	RBM15	TAL2	ZFP36L1
BCOR	DNMT1	GATA1	KLF5	NFIB	RBMX	TBX18	ZFX
BRCA1	DNMT3A	GATA2	KLF6	NFKB2	REL	TBX22	ZHX2
BRCA2	DOT1L	GATA3	LDB1	NFKBIA	RUNX1	TBX3	ZIC3
BRD7	EED	GLI3	LMO1	NONO	RUNX1T1	TCEA1	ZIM2
BRD8	EGR2	GTF2I	LMO2	NOTCH2	RXRA	TCEB1	ZNF208
BRIP1	ELAVL2	HDAC9	LMX1A	NOTCH3	SALL3	TCERG1	ZNF226
BRPF3	ELF3	HEY1	LYL1	NPM1	SATB2	TCF12	ZNF331
BTG1	ELF4	HIST1H1B	LZTR1	NR3C2	SETBP1	TCF3	ZNF384
BTG2	ELK4	HIST1H1C	MAF	NR4A3	SFPQ	TCF7L2	ZNF469
CBFA2T3	ELL	HIST1H1D	MAFA	NSD1	SIN3A	TFAP2D	ZNF595
CBFB	EP300	HIST1H1E	MAFB	OLIG2	SMAD2	TFDP1	ZNF638
CDX2	EPC1	HIST1H2BC	MAML1	PAX3	SMAD4	TFE3	
CDX4	ERCC2	HIST1H2BD	MAX	PAX5	SMARCA1	TFEB	

^1^ This list is obtained by crossing data from the known and candidate cancer genes lists (http://ncg.kcl.ac.uk/statistics.php) with the list of known human transcription factors [5]).

**Table 2:** Examples of polyamides targeting oncogenic transcription factors.

Transcription Factor Family	Transcription Factor Target	Tumor Model	References
AT-rich binder	TBP	-	[323]
	LEF-1	Colon	[324]
Hormone/Steroid receptor	estrogen receptor	Breast	[325]
	androgen receptor	Prostate	[326,327]
	glucocorticoid receptor	-	[327,328]
B-Zip	GCN-4	-	[328,329]
	AP-1	-	[330,331,332]
ETS-domain	ETS-1	-	[333,334]
	EVI1	Leukemia	[335]
	ELK-1	Breast	[336]
	PU.1/SPI1	-	[337]
Zn-Finger	TFIIIA	-	[338,339]
	Zif268	-	[340]
Others	NF-Y	Lung	[341,342,343,344,345]
	NFκB	Lung, Osteosarcoma	[346,347]
	HIF1	Kidney, Glioblastoma, Multiple myeloma	[348,349,350,351]
	c-MYC	Osteosarcoma, Burkitt’s lymphoma	[352,353]
	OCT1	Prostate	[354]
	RUNX	Acute myeloid leukemia	[355]
	E2F1	Chronic myeloid leukemia	[355]

**Table 3:** Examples of mithramycins deregulated genes that occurs upon inhibition of SP1 transcription factor.

Type of Protein	Gene Promoter	Tumor Model	References
Transcription factors	c-MYC	Cervix carcinoma	[386]
	FoxM1	Liver	[387]
	KLF5	Breast	[388,389]
	SNAI1	Salivary adenoid cystic carcinoma	[390]
	SP1		
Regulators of cell proliferation	CDKN1A	Liver	[391]
	Survivin	Colon	[392]
	Ki-67	-	[393]
	CRABP1	Ovary	[394]
Tumor suppressors	KCNMA1	Ovary	[394]
	p73	Lung	[395]
Apoptotic genes	XIAP	Ovary	[394]
	BAK1	Liver	[396]
Secreted factors	Collagen-alpha 1	-	[397]
	Tissue factor	-	[397]
	MUC2	Colon	[398]
	TGFBI	Lung, Breast	[399]
	ECRG4	Leukemia	[400]
Membrane associated proteins (receptors, transporters)	Androgen receptor	Prostate	[401]
	ABCG2	Lung	[402]
	FZD1	-	[403]
Metabolic enzymes	Dihydrofolate reductase	Breast	[404]
	Carbonic anhydrase IX	-	[405]
Cell differentiation	MSI2	Lung	[406]
Cell movement	KIF2C kinesin	Colon	[407]
